# Nutritional Care for Patients with Ebola Virus Disease

**DOI:** 10.3201/eid2601.191024

**Published:** 2020-01

**Authors:** Mija Ververs, Magi Gabra

**Affiliations:** Centers for Disease Control and Prevention, Atlanta, Georgia, USA

**Keywords:** Ebola, Ebola virus disease, nutrition, nutritional care, Ebola treatment, Ebola virus infection, viruses, DRC, West Africa

## Abstract

During the Ebola virus disease (EVD) outbreak of 2014–2016 in West Africa, practitioners faced challenges providing nutritional care for patients in Ebola treatment units (ETUs). The current EVD outbreak in the Democratic Republic of the Congo demonstrates the need to understand lessons learned from previous outbreaks and to update nutritional guidelines. We conducted a literature review to identify articles that included nutrition as an integral part of supportive care. We found little information on the specific nutritional care or practical challenges within an ETU. This review showed that nutritional care for EVD patients is poorly described, and therefore the optimal composition and implementation of nutritional care remain unknown. We recommend that researchers and practitioners share specific and practical details of their experiences in providing nutritional support within ETUs to expand the knowledge base and ultimately improve the nutritional care for an increasingly prevalent patient population.

In 2014, the World Health Organization (WHO), the United Nations Children’s Fund (UNICEF), and the World Food Programme (WFP) produced interim guidelines with recommendations for providing nutritional support to patients in Ebola treatment units (ETUs) (*1*). These guidelines were based on existing WHO evidence-based guidance adapted to the Ebola crisis, in addition to a rapid literature review on Ebola virus disease (EVD) and nutritional management of hemorrhagic fevers. The document aimed to address key clinical problems for EVD patients, their nutritional needs, and optimal nutritional care, including the practical aspects of providing nutritional support within treatment centers.

In the context of the current Ebola virus outbreak in the Democratic Republic of the Congo (DRC), we summarize existing practices and research findings on nutritional care for EVD patients in ETUs and compare them, when relevant, with 2014 WHO/UNICEF/WFP guidelines. More specifically, we aim to identify gaps to guide future practices and research, ultimately leading to improved nutritional guidelines.

## Methods

We conducted a literature search using the MEDLINE database (through PubMed and OVID search engines), Global Health, and Scopus, and used Google Scholar to search gray literature. Medical Subject Headings terms used included “hemorrhagic fever, Ebola,” “Ebola virus,” and “nutrition.” We also searched on the following terms: diet, vitamin, malnutrition, breastfeed, nutrients, fortified, micronutrient, caloric, calories, soup, porridge, cereal, legume, sugar, and dextrose. The search identified articles relating to Ebola virus and nutrition published from January 1, 2014, through August 30, 2019. We did not apply language restrictions, and for search engines that allowed it, we included only articles about studies with human subjects. We screened only the first 120 results from Google Scholar because of decreasing relevance of articles. We downloaded and managed articles through EndNote X9 (Clarivate Analytics, https://endnote.com).

## Results

We identified a total of 429 articles (Figure); 268 articles remained after deduplication. We excluded 240 articles after screening for information on nutrition or feeding during care for Ebola patients; content that caused exclusion included animal and bushmeat consumption in relation to Ebola transmission, testing and safety of breast milk in seropositive patients, food availability, malnutrition, agricultural stability before and during Ebola outbreaks, pharmacologic experiments for Ebola treatment, and molecular studies on the pathogenesis of Ebola virus. We completed full-text review on the remaining 28 articles. We excluded 5 non-English articles and added 1 more article identified from gray literature, resulting in review of 24 articles.

**Figure:**
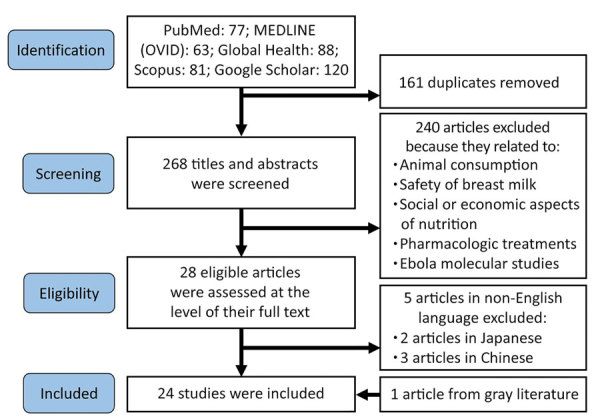
Flowchart summarizing literature search and selection process for review of nutritional care for patients with Ebola virus disease.

Most of the articles (n = 14) on nutritional support during Ebola treatment were case studies, cohort studies, or management recommendations focused on the clinical features, care provided, and outcomes observed (*2*–*15*). Four articles focused solely on patient care provided in the United States and Europe (*5*,*6*,*8*,*14*). Six described patients in West Africa during the 2014 outbreak: 3 in Sierra Leone (*3*,*4*,*11*), 1 in the Democratic Republic of the Congo (*9*), 1 in Liberia (*2*), and 1 in both Liberia and Sierra Leone (*15*). One particular study was a prospective cohort study on the effects of supplemental vitamin A on disease mortality rate (*15*). Two articles were field manuals, one an emergency interim country guidance for clinical management (*16*) and the other a list of essential medications for Ebola patient care (*17*). One article provided evidence-based guidelines for patient care using the Grading of Recommendations Assessment, Development, and Evaluation (GRADE) methodology (*18*). One was a systematic review of current trends of Ebola virus management (*19*). Two articles were analyses of policies and programming regarding infant feeding (*20*) and nutrition (*21*). An editorial on nutritional management of patients with Ebola virus disease (*22*) and a comment published on the overall changes in care for Ebola virus disease (*23*) were included, as well as a personal report of a nurse’s experience in the field while working in an ETU (*24*). The final item was a letter from a nutrition advisor during the Ebola outbreak (*25*), which we identified from gray literature.

Despite the initially large number of papers with the key search term “nutrition” related to EVD case management, few explicitly described the details of the delivered nutritional support. Most clinical management papers delineated supportive care for dehydration and electrolytes through oral rehydration solutions (ORS) or intravenous fluid administration and did not refer to nutrition. Two studies and a retrospective review concluded that dehydration was associated with worse outcomes and increased mortality rates in patients (*7*,*9*,*11*). Both Leligdowicz et al. (*7*) and Smit et al. (*11*) included electrolyte imbalance as an additional risk factor for poor outcomes. Smit et al. found that poor nutritional status was also associated with increased mortality (*11*). The systematic review by Sivanandy et al. noted that nutritional care should include a “good amount of protein supplements” but did not specify a recommended amount or type of protein (*19*).

The evidence-based recommendations produced by Lamontagne et al. supported the importance of hydration in supportive care and monitoring serum biochemistry for electrolyte repletion (*23*). In creating the recommendations, a multidisciplinary panel met to analyze data on supportive care and voted to create a list of evidence-based guidelines. The panel strongly recommended administering ORS in an adequate amount, rather than using nonstandardized rehydration. The panel also strongly recommended that serum biochemistry (e.g., testing of electrolytes, glucose) should be made available, but the group did not produce any statements on nutrition (*18*).

### Clinical Cases

Johnson et al. (*5*) and Uyeki et al. (*14*) described total parenteral nutrition (TPN) in patient care in the United States and Europe. Patients treated in the biocontainment unit at the University of Nebraska Medical Center had a “moderate state of protein malnutrition” at admission, but protein malnutrition was not defined (*5*). A nutritional therapist was consulted for both cases and TPN was started, but further details were not provided. In another case study, a patient treated in Hamburg, Germany, did not tolerate enteral nutrition, and so the treatment team initiated TPN (*6*). When the patient showed improvement, enteral nutrition was reinitiated with a low-fiber standard formula; the authors did not provide details of the diet. A physician turned patient hydrated himself with a commercial sports rehydration beverage and a sugary drink mix before seeking treatment at Emory University Hospital in Atlanta, Georgia, USA, where he was given protein drinks and multivitamins. No information on the content of the protein drink or the frequency of use was provided (*8*). Similarly, a case study of 581 patients in an ETU in Freetown, Sierra Leone, documented the use of 1 capsule of ImmunoBoost nutrition supplement (Novopharm Formulations, http://www.novopharm1.com) per day and ORS and juice drinks consumed freely (*3*). However, the authors did not describe treatment rationale or composition.

In a recent study, Aluisio et al. assessed oral vitamin A supplementation in 424 patients admitted to ETUs in Sierra Leone and Liberia in 2014–2015 (*15*). Mortality rate was significantly lower among patients who had received 200,000–400,000 IU of vitamin A in the first 48 hours of admission, compared with those who had not received vitamin A at admission (Relative Risk = 0.77, 95% CI 0.59–0.99; p = 0.041). An editorial published in the Asian Pacific Journal of Tropical Disease provided specific recommendations on the energy, protein, fat, and micronutrient components for the nutritional management of EVD patients (*22*). The authors recommended the following: achieving a protein requirement of 1.2 g/kg of ideal bodyweight, 55%–60% of total energy needs via carbohydrates, provision of soft foods to avoid gastrointestinal irritation, and avoidance of trans-fatty acids. It is important to note that there were no references to clinical research or case studies of patients with EVD to support these recommendations.

A nurse’s account of her experience working in an ETU in Liberia provided some insight on actual feeding practices (*24*). Wilson reported that a locally popular artificial juice powder composed of mostly sugar and vitamin C (Foster Clark, http://fosterclark.com) was occasionally mixed with the ORS to increase palatability. In addition, patients received 3 meals, individually packaged in plastic bags, daily. Patients who were well enough often consumed food purchased or cooked by family or loved ones.

### Pediatric Nutrition

Trehan et al. published 2 papers with extensively detailed nutritional care specifically for pediatric patients with Ebola virus disease (*12*,*13*). Recommendations included, though were not limited to, assessing nutritional status using mid-upper arm circumference, providing ample ORS and therapeutic milks (F-75, F-100; Nutriset, https://www.nutriset.fr), and serving ready-to-use therapeutic foods (RUTF) in biscuit and paste form. To improve hydration and intake, flavored ORS solutions were recommended if available, although drinks with added sugar were not recommended because they may exacerbate diarrhea. RUTF pastes and biscuits were 2 examples of energy-dense foods with complete micronutrient and macronutrient composition that retain taste and cleanliness for extended periods and are easily consumed by weak patients. The authors recommended the prioritization of RUTF over local foods in the acute phase of the illness. Instructions were included on how to prepare RUTF to produce a semi-solid porridge for easier consumption and described the minimum quantity of RUTF (for children <15 years varying from 2 to 5 sachets with RUTF paste or 3 to 8 RUTF biscuits). For infants <6 months of age, the authors recommended ready-to-use infant formula. For children >6 months of age who were only able to tolerate liquids, the authors recommended therapeutic milks over dairy milk or commercial infant formulas because of the increased nutritional content. Although it was more nutritionally complete than F-75, F-100 therapeutic milk was not endorsed for all pediatric patients because its higher osmotic load compared with that of F-75 may cause increased diarrhea. The authors recommended that formulas be refrigerated and prepared multiple times a day to avoid spoiling.

A nutrition advisor described infant feeding methods to reduce mother-to-infant transmission (*25*). Because feeding utensils and human-to-human contact carried an increased risk for transmission, the author reported the use of a syringe attached to a feeding tube to safely feed infants. Brandt et al. reviewed infant feeding policies and programming during the 2014–2016 Ebola outbreak and highlighted the inconsistencies of the messaging around breastfeeding (*20*). They recommended including infant and young child feeding experts in outbreak response and creating consistent, appropriate, and tailored messages regarding breastfeeding and infant nutrition in the early phase of the response. 

### Perception of Nutrition Response

One descriptive qualitative study of key informants analyzed community perceptions of the nutrition-related response in Guinea (*21*). That study had 2 main objectives: the first was to determine how the Ebola outbreak affected infant and child nutrition on a community level, and the second was to gauge stakeholders’ perception on the acceptability and effectiveness of the nutrition response, including the WHO/UNICEF/WFP 2014 interim guidelines for nutritional care. A consistent theme across informants was the lack of emphasis on nutrition by health professionals and community members during the Ebola outbreak. Key informants also noted limitations around the use of the WHO/UNICEF/WFP interim guidelines; some informants were unaware of the guidelines or questioned their usefulness, and some reported finding the guidelines useful but difficult to implement. The authors recommended that nutrition be a core component of response and integrated into all aspects of care, treatment, and recovery.

## Discussion

In this literature review, we summarize existing practices and research findings on nutritional care for EVD patients. A wealth of literature exists on the clinical management and supportive care, but our review found only a limited number of publications on specific nutritional care, and they often lacked detailed descriptions of the actual nutritional care provided. The results could lead to the conclusion that the role of nutritional care in ETUs is perceived to have limited importance. However, the importance of good nutrition in fighting infection is widely known, and several researchers emphasized the need for good nutritional care for EVD patients (*11*–*14*,*17*,*21*,*22*). In addition, it is well established that adequate nutrition is essential in the management of critical illness or sepsis (*26*,*27*). Our review showed that nutritional care is poorly described and therefore the optimal composition and implementation of care remains unknown. One noticeable finding was the use of total parenteral nutrition (TPN) only in high-resource settings. It is unclear whether TPN is preferred over enteral nutrition and whether the role of enteral nutrition for patients’ survival is different in high- or low-resource settings.

We intended to compare actual nutritional care in ETUs with the 2014 WHO/UNICEF/WFP interim guidelines. However, various care descriptions from our literature review preceded the availability of these guidelines (*2*,*3*,*5*,*6*,*8*,*9*,*14*,*17*,*22*,*24*). This restricted our ability to compare recommended and actual practices. Only 3 works published after November 2014 commented on using the guidelines or reported any successes or challenges with attempting to follow them (*12*,*13*,*21*).

WHO recommends that EVD patients should be provided with a minimum recommended daily allowance (RDA) of nutrients through normal traditional or fortified foods (*1*,*16*) or micronutrient powders (*28*). It also states that, until further evidence is available, excess use of any micronutrient for EVD patients is not recommended, unless correcting for a specific micronutrient loss (e.g., treating hypokalemia). However, in many EVD-affected countries in sub-Saharan Africa, malnutrition, including micronutrient deficiencies, is widely prevalent (*29*,*30*), and 1 RDA of, for example, vitamins would not sufficiently address existing suboptimal levels in EVD patients.

To improve nutritional care for EVD patients, more documentation is needed on nutritional care in ETUs. We recommend that researchers and practitioners share specific and practical details of their experiences in providing nutritional support within ETUs to further facilitate the scientific base and ultimately improve the nutritional care for an increasingly prevalent patient population. In addition, research is necessary to determine whether specific macronutrients or micronutrients improve treatment outcomes in ETUs and elucidate their mechanism of action. For instance, the roles of albumin (*14*), selenium (*31*,*32*), and electrolytes (*33*,*34*) deserve further exploration, and the recognized importance of vitamin A in mounting an immune response to infectious diseases urgently merits further studies. It is also worth investigating whether anthropometric changes in patients admitted to ETUs relate to outcomes of illness and death. In addition, we see a need to examine whether the recommendation of providing 1 RDA is sufficient for managing patients with nutrient losses secondary to EVD-induced enteropathy, complicated by an underlying suboptimal nutritional status. We further underline the need for immediate research on breast milk and transmissibility of EVD (*35*). Last, we propose to not only examine whether specific nutritional care can raise patient survival rates, but to assess how it may contribute to symptom relief in the critically ill. Although adequate nutrition cannot cure patients with EVD, maintaining an optimal nutritional status could improve their response to treatment.
